# Defect‐Symmetry Engineered Gyroidal Photonic Crystals Achieve Monotonic Modulation of Bandgap Width With a Stable Mid‐Gap

**DOI:** 10.1002/advs.78055

**Published:** 2026-09-29

**Authors:** Shuo Huang, Yang Zhou, Wenxin Ning, Hengliang Wang, Lu Han, Xueyan Feng

**Affiliations:** ^1^ State Key Laboratory of Molecular Engineering of Polymers Department of Macromolecular Science Fudan University Shanghai China; ^2^ Department of Physics Fudan University Shanghai China; ^3^ School of Chemical Science and Engineering Tongji University Shanghai China

**Keywords:** 3D photonic crystal, defect engineering, microstructure fabrication, monotonic modulation, photonic bandgap

## Abstract

Three‐dimensional (3D) photonic crystals with a photonic bandgap (PBG) facilitate strong control of light propagation. In many applications, PBG width needs to be systematically manipulated while the system needs to be operated at predetermined wavelengths. Conventional methods for such situations require the coordinated modulation of multiple factors, which complicates practical implementation. Achieving monotonic tuning of the PBG width while maintaining a stable central frequency through single‐parameter modulation remains challenging. Here, inspired by the twin grain boundary discovered in natural gyroidal networks, we construct a series of gyroid‐derived tubular networks incorporating twin mirror symmetry as a periodic design element with different gap distances between the twin mirror planes. Both calculations and experimental validation demonstrated that these structures exhibit monotonic tunability in PBG width with increasing gap distance between the twin mirror planes, while the central frequency of the PBG maintains stable. Electric field analysis indicates that the introduced mirror symmetry with different gap distances changes the distribution of electrical field from discrete mode into continuous mode gradually, contributing to the PBG monotonic phenomenon. This work opens a route for manipulating the spectral position and width of PBG in 3D photonic crystals separately and highlights the potential of structural defect engineering for photonic crystal design and fabrication.

## Graphical abstract

Inspired by the twin grain boundary discovered in natural gyroidal networks, a series of gyroid‐derived photonic crystals are constructed based on defect‐symmetry engineering. The new series of structures enables monotonic tuning of the width of the photonic bandgap (PBG) while the central frequency of the PBG maintains stable. This study provides a new strategy for tailoring the PBG of three‐dimensional photonic crystals.

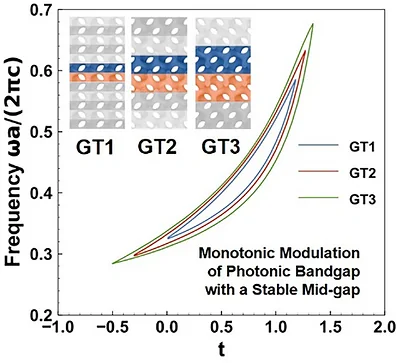

## 1 Introduction

Three‐dimensional (3D) photonic crystals are 3D periodic dielectric media with a photonic bandgap (PBG) [1]. In photonic crystals possessing a complete PBG, electromagnetic wave propagation within the forbidden frequency range is prohibited in all directions. Such light‐manipulation capability facilitates diverse applications, including high‐performance optical filters, passive radiative cooling, and the realization of photonic quantum computing, etc [2, 3]. Among the key characteristics of photonic crystals, the spectral position and width of the PBG are fundamentally important, as they determine both the operational frequency and functional bandwidth of the corresponding photonic devices. Tremendous efforts have been made to manipulate these two characteristics of PBG [4, 5, 6]. In many applications such as passive radiative cooling [7, 8, 9], optical communication [10, 11], and laser systems [12], bandgap width needs to be systematically manipulated while the systems need to be operated at predetermined wavelengths. Conventional methods for such situations require the coordinated modulation of structural type/dimension, dielectric constant, dielectric filling fraction, etc. Such coupling complicates practical implementation. Realizing monotonic control of the PBG width with a stable central frequency through single‐parameter tuning is still challenging.

Structural type, structural size, and dielectric contrast are the key factors influencing the PBGs [5]. Different structures inherently possess distinct band structures. Such variability intrinsically makes simply changing the structure type is not an ideal way to systematically manipulate the PBG width and position, particularly in the large‐scale design and fabrication of photonic devices. For the same structures, monotonic tuning of the bandgap width at a stable central frequency through single‐parameter modulation is also challenging. Traditional approaches for modifying the PBG width typically induce a shift in its spectral position. Preserving a constant frequency generally necessitates coordinated variations of several structural and material parameters, such as the characteristic dimensions, dielectric filling fraction, and dielectric constant. Therefore, to achieve monotonic control of the bandgap width while remaining at a stable central frequency through single‐parameter tuning, the design and development of new systems is highly demanded.

Since the seminal proposal of the photonic crystal concept by Yablonovitch [1], researchers have identified various structures with robust complete PBGs such as woodpile [13], diamond [14], gyroid [15], and inverse opal structures [16, 17, 18, 19], etc. These designs are frequently inspired by natural minerals or biological organisms. Researchers are also exploring innovative perturbations of classical structures, including shifted double gyroid [20], non‐affinely transformed gyroids [21, 22], and even amorphous diamond architectures [23]. These structural evolutions expand new degrees of freedom for light manipulation and next‐generation photonic materials. Beyond the classical morphologies and corresponding deformations, structural defect engineering, which is based on manipulating the features of structural defects within established structures, is increasingly being involved into structure design [24]. For example, the utilization of point defects to achieve the spatial localization of electromagnetic modes and the incorporation of line defects to facilitate waveguiding have demonstrated the ability to manipulate material photonic behaviors via structural defect engineering [25]. The conventional research in this area has mainly focused on the integration of such localized defects to break the symmetries of established and perfect crystalline frameworks [25, 26, 27, 28, 29, 30]. However, leveraging the intrinsic symmetry of the structural defects as a periodic design element to generate new photonic crystal structures has not been explored.

The gyroid is one of the most notable morphologies for photonic crystals [2, 15, 17, 26, 30, 31, 32, 33]. It is a 3D tubular network structure with a considerable PBG. Previous studies have indicated that twin boundaries, which are related to additional mirror symmetry, are frequently encountered during the self‐assembly of gyroid structures in different systems [34, 35, 36]. Here, we exploit such twin defect‐associated mirror symmetry as a periodic symmetry element to construct a series of gyroid‐derived tubular network structures with increasing gap distance between the parallel twin mirror planes (named GT1, GT2, and GT3). This defect‐symmetry‐driven design enables decoupled control of PBG width and frequency. Calculations based on the plane‐wave expansion (PWE) method reveal that these structures with the same size, same volume fraction, and same material dielectric contrast exhibit monotonic increases in relative PBG width (9.2%, 13.2%, and 17.9% for GT1, GT2, and GT3, respectively), while the central frequency of the PBG varies by less than 0.85% relatively, remaining nearly unchanged. Such photonic behavior is further experimentally validated by measurement of corresponding photonic crystal devices fabricated by two‐photon lithography (TPL) and atomic layer deposition (ALD). Electric field analysis indicates that the introduced twin mirror symmetry with different gap distances changes the distribution of electrical field from discrete mode into continuous modes gradually, which is the key factor contributing to the PBG behaviors. This work establishes a general framework for decoupled bandgap engineering in 3D photonic crystals and highlights the potential of defect‐symmetry‐derived architectures for photonic crystal design and fabrication.

## 2 Results and Discussion

### 2.1 Design of Photonic Crystals

In our previous study, a twin boundary along the (422) lattice plane in a block copolymer assembled double‐gyroid (DG) material was characterized via 3D electron microscopy tomography (Figure S1). The detailed morphological and topological features of the twin boundary have been revealed, and the results match well with the twin model constructed based on the level‐set model [34]. Such twin boundaries provide additional mirror symmetry to the gyroid networks. In the real sample, the twins tend to appear in groups in the form of parallel 2D twin planes. The gap distance between these parallel planes would vary (in the value of $n\frac{\sqrt{6}}{6}l$, where *l* is the cube unit cell length, *n* is an integer value, and $\frac{\sqrt{6}}{6}l$ is the gap between two adjacent (422) planes, as shown in Figure 1A). The morphology within the gap still remains as a typical ordered gyroidal network. The DG structure is composed of two interpenetrating and independent gyroid networks. Inspired by the twin phenomena in DG, we focused on one of the two interpenetrating networks (i.e., a single gyroid, SG), extracted the structural parts between two twin planes with different gap distances ($n\frac{\sqrt{6}}{6}l$) as structural motifs, mirrored the motifs periodically, and devised a new class of gyroid twin (GT) tubular network structures. The details are as follows:

**FIGURE 1:**
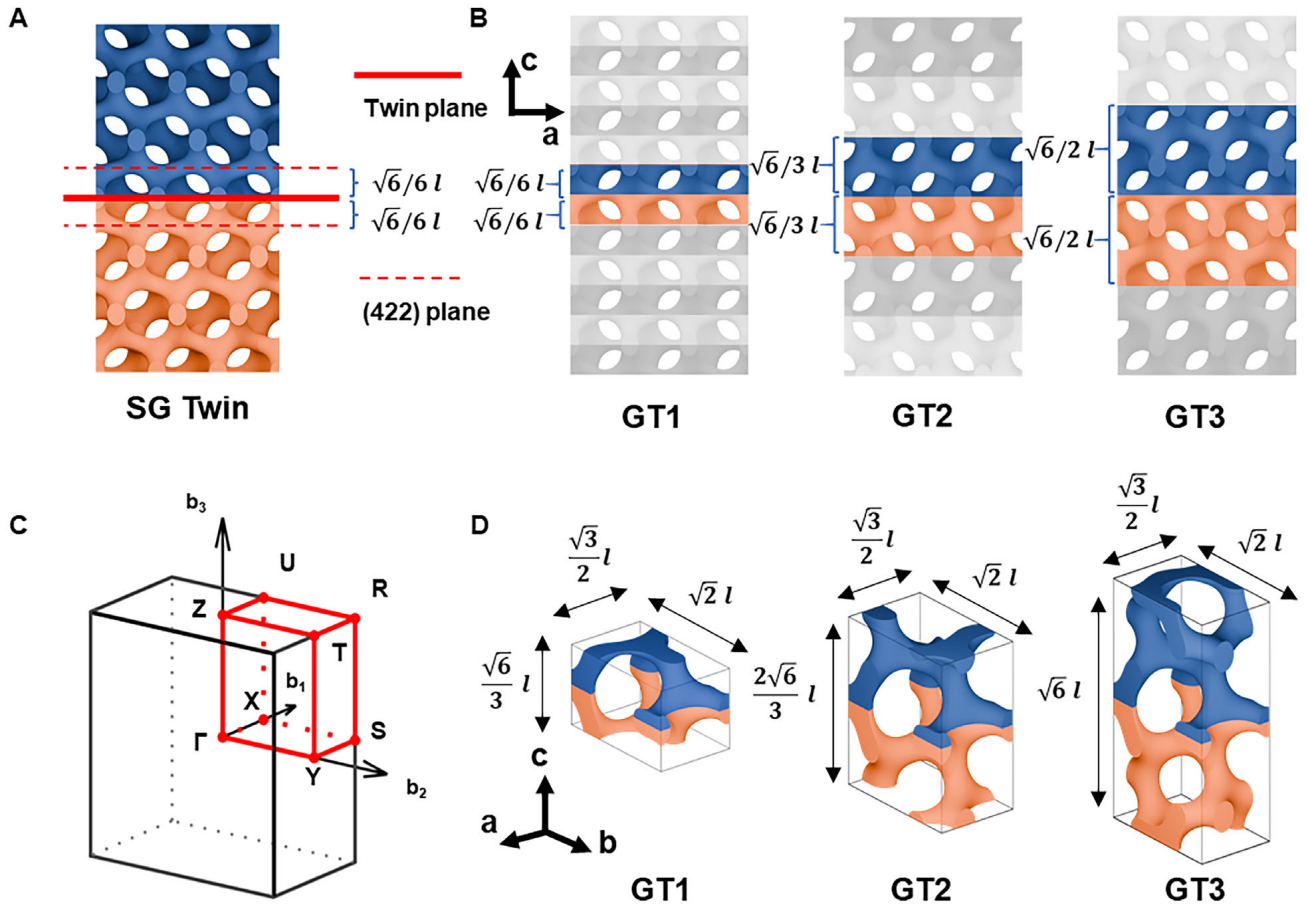
Construction of twin‐symmetry‐derived gyroidal tubular network photonic crystals (GT1‐GT3). (A) SG twin model with the (422) plane as the twin plane. The gap between two nearest (422) planes is $\frac{\sqrt{6}}{6}l,$ while *l* is the gyroid cube unit cell length. (B) GT1, GT2, GT3 tubular networks, the smallest repeated parts within GT1 to GT3 are highlighted in color, the orange parts and blue parts are mirrored by the (422) plane. (C) The Brillouin zone of orthorhombic lattices. The red lines represent the paths of the first Brillouin zone. (D) The unit cells of GT1 to GT3.

An SG structure (space group of *I*4_1_32) with a cubic lattice parameter *l* was first constructed by the level‐set equation:

$$\sin\left(2\pi x\right)*\cos\left(2\pi y\right)+\sin\left(2\pi y\right)*\cos\left(2\pi z\right)+\sin\left(2\pi z\right)*\cos\left(2\pi x\right)=t$$

*t* is a constant that is related to the volume fraction of the tubular network, as shown in Figure S2.

Thus, the SG segments, located between two parallel twin planes with different gap distances of $\frac{\sqrt{6}}{6}l$, $2\times \frac{\sqrt{6}}{6}l,$ $3\times \frac{\sqrt{6}}{6}l$ are cropped out and mirrored periodically to generate the GT1, GT2, and GT3 tubular network structures, respectively (Figure 1B). Notably, though these new structures are derived from the SG network, they are intrinsically different from SG. For SG structures, they are chiral 10–3^10^ tubular networks [34]. The smallest repeatable closed loop in the SG network consists of 10 nodes, with each node having three neighbors. For GT structures, they are achiral tubular networks, consisting of nodes with 3‐, 4‐ connections (f3 nodes and f4 nodes, respectively). The detailed loop analysis of GT1 to GT3 is summarized in the smallest loop analysis in Figure S3.

For GT1 to GT3, they all belong to orthorhombic crystal symmetry, with corresponding orthorhombic Brillouin zones, as shown in Figure 1C. To facilitate subsequent photonic characterization, the unit cells of GT1 to GT3 are extracted. For $\overset{⇀}{a}$ & $\overset{⇀}{b}$ unit cell vectors within the twin plane, they remain the same for GT1 to GT3 (i.e., $|\overset{⇀}{a}|=\frac{\sqrt{3}}{2}\;l,\;\left|\overset{⇀}{b}\right|=\sqrt{2}\;l$ as shown in Figure 1D and Figure S4). For $\overset{⇀}{c}$ unit cell vector, $|\overset{⇀}{c}|=\frac{\sqrt{6}}{3}\;l$, $\frac{2\sqrt{6}}{3}\;l$, $\sqrt{6}\;l$ for GT1, GT2, GT3, respectively (Figure 1D).

### 2.2 Calculation of Photonic Band Structures

The photonic band structures of GT1 to GT3 tubular networks were calculated via the PWE method, using the MIT Photonic Bands (MPB) open‐source software package [37]. For the calculated *k*‐points, the schematic paths along the boundary of the irreducible Brillouin zone, as shown in Figure S5, were chosen. The dielectric constant was set as ε  =  12, similar to Si. The level‐set model constant *t* was set as 0.9, and the gyroid cubic unit cell length *l* was set as 1 for all structures.

As the band structures shown in Figure 2A–C, GT1, GT2, and GT3 all have sizeable complete PBGs (the redundant paths are shown in Figure S5). The gap ranges are 0.447–0.490 for GT1, 0.437–0.499 for GT2, and 0.423–0.506 for GT3. The complete PBG broadens with increasing spacing of the extracted basic motif between two mirror twin planes. In addition, the mid‐gap frequencies ω_*m*_ remain stable for GT1, GT2 and GT3. To further investigate this phenomenon, the band structures of GT1 to GT3 with different level‐set constant *t*, corresponding to the volume fraction of the tubular networks, were calculated. The lower and upper band edge frequencies of the PBG were extracted (Figure 2D). The results indicate that the bandgap behaviors of GT1, GT2, and GT3 with different *t* values exhibit the same trends as introduced below. As *t* increases, the PBG width Δω initially increases and subsequently decreases, reaching a maximum at *t* ≈ 0.9. Meanwhile, the PBG central frequency ω_*m*_ keeps increasing monotonically with *t*. Notably, from the appearance to disappearance of PBG, the ω_*m*_ values of GT1 to GT3 always remain close at each *t* value point. As Maxwell's equations are scale invariant, the relative gap is defined as Δω/ω_*m*_ to facilitate comparison among different photonic crystal structures. From GT1 to GT3, the relative gap increases significantly, which exhibits good tunability. For example, at *t*  =  0.9, the relative gaps are 9.2% for GT1, 13.2% for GT2, and 17.9% for GT3, while the central frequency of the PBG remains almost unchanged (the variation is less than 0.85%). Since $\sqrt{6}l$ is the repeated length perpendicular to (422) twin plane (along <211> direction) of SG, GT4, GT5, and GT6, with corresponding twin plane gap distances of $4\times \frac{\sqrt{6}}{6}l$, $5\times \frac{\sqrt{6}}{6}l$, $6\times \frac{\sqrt{6}}{6}l$, respectively, were also constructed and evaluated. The results indicate that the ω_*m*_ values for GT4, GT5, GT6 still remain stable, while the gap ranges exhibit only minor variations (Figure S6). Compared with the original SG (Figure S7), the GT photonic crystals exhibit markedly different photonic band structures. Although the constituent segments of the GT structures are derived from SG, the introduced mirror symmetry substantially alters their overall symmetry and structural topology. From GT1 to GT3, an increasing proportion of the original SG structure is retained, and the corresponding band characteristics progressively approach those of the original SG, while distinct differences remain.

**FIGURE 2:**
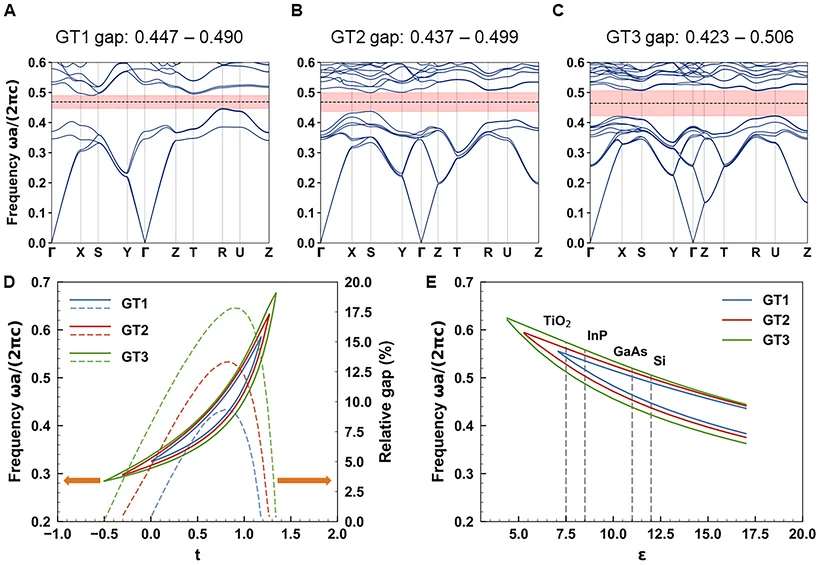
Analysis of photonic band structures of GT1 to GT3. (A–C) The band diagrams of GT1 to GT3 (the blue lines) along the paths of the first Brillouin zone. The frequency ω is normalized as $\frac{\omega a}{2\pi c}$, *a* is set the same for GT1 to GT3 (*a* = 1), c is the speed of light. The complete PBG are shown as red regions, and the dashed lines indicate the central frequency of the PBG. (D) The solid lines with different colors indicate the lower and upper band‐edge frequencies of the PBG of GT1 to GT3 at different level‐set model constant *t*. The relative gap values for GT1 to GT3 are plotted with dashed lines. (E) The band‐edge frequencies of PBG of GT1 to GT3 at different dielectric constants.

To study the generality of the photonic property discovered above, the band structures of GT1 to GT3 with different dielectric constants (ε) were further calculated, as shown in Figure 2E. As ε gets larger, the Δω of GT1 to GT3 increases and ω_*m*_ decreases, which is a general phenomenon in photonic crystals. Still, throughout the calculated ε range which covers typical photonic crystal materials, such as Si (amorphous silicon, ε ≈ 12), GaAs (zinc blende, ε ≈ 11), InP (zinc blende, ε ≈ 8.5), TiO_2_ (rutile, ε ≈ 7.5), the PBG width Δω increases monotonically from GT1 to GT3, while the central frequency of the PBG ω_*m*_ remains stable at each ε value.

### 2.3 Fabrication of Photonic Crystal Devices

To experimentally validate the photonic crystal phenomena observed above, corresponding infrared photonic crystal devices were fabricated (Figure 3A). Two‐photon lithography was first employed to print the initial cross‐linked acrylate‐based polymer scaffold. To open a PBG, materials with a high refractive index are necessary. Here, the atomic layer deposition method was used to coat high refractive index materials onto the low refractive index polymer. To ensure the high‐fidelity preservation of the printed structure, which is critical for the resulting optical response, low‐temperature atomic layer deposition for TiO_2_ deposition was employed. This approach effectively mitigates structural deformation during processing [13, 16, 38, 39], which makes the fabricated structures have a great agreement with the designed models, as shown in Figure S8, ensuring that the final photonic crystals accurately preserve the intended structural design.

**FIGURE 3:**
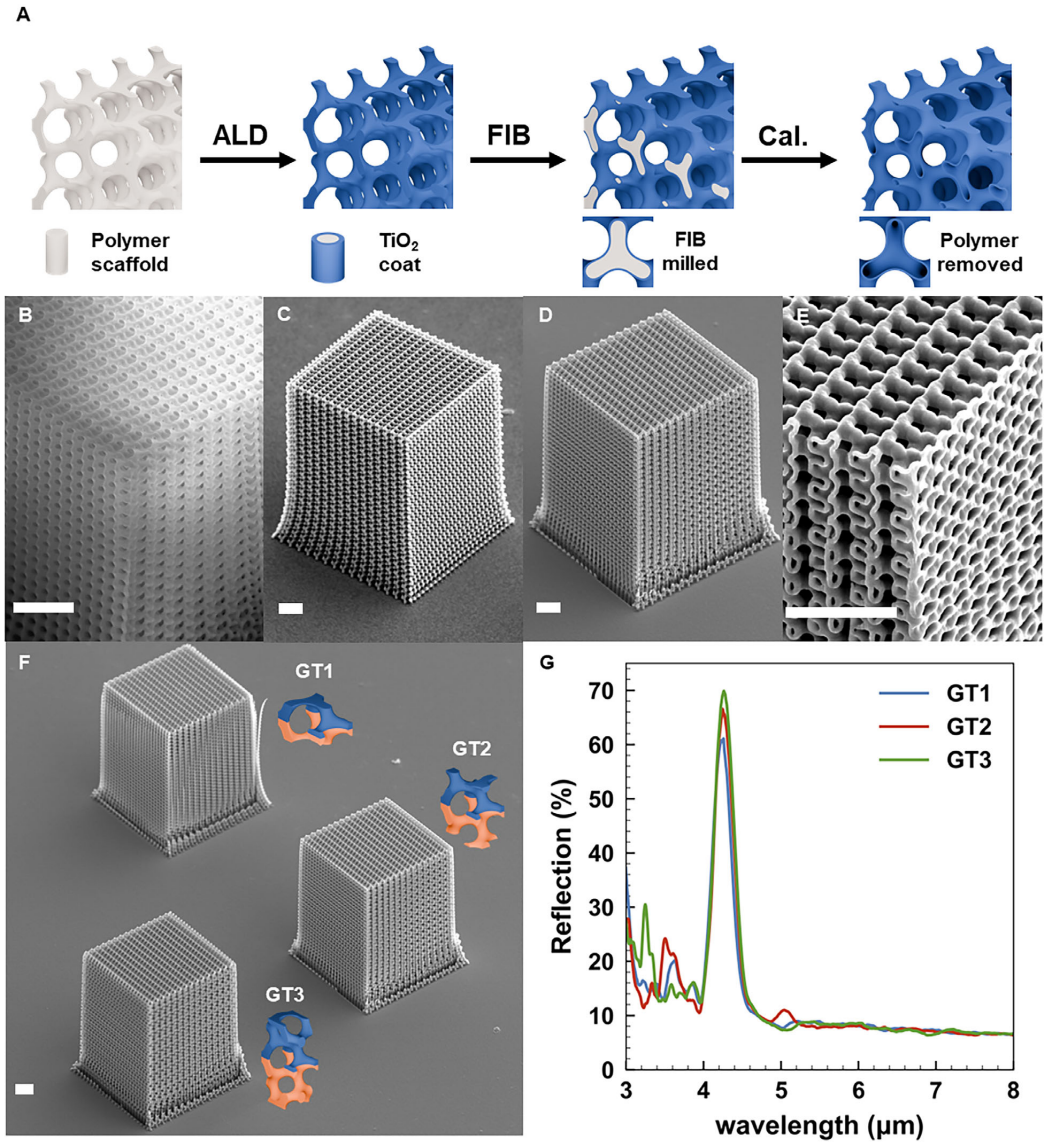
Fabrication and spectra characterization of the designed GT1 to GT3 photonic devices. (A)Schematic illustration of the fabrication process for the photonic devices. (B–E) Representative SEM characterization of each step during the fabrication process. (F) SEM characterizations of the final GT1 to GT3 photonic devices with corresponding single unit cell models. (G) Reflectivity spectra of GT1 to GT3. Scale bar: 7.5 µm.

Based on the unit cell parameters of GT1, GT2, GT3 introduced above, with an original single gyroid cubic unit cell length *l*  =  2.50 µm, the unit cell length $|\vec{a}|$, $|\vec{b}|$ were kept identical for GT1 to GT3 ($|\vec{a}|$= 2.17 µm, $|\vec{b}|$= 3.54 µm), and the unit cell length $|\vec{c}|$ for GT1, GT2, and GT3 are 2.04, 4.08, 6.12µm, respectively. The polymer scaffold was first prepared (Figure 3B), and to maintain an identical overall device dimension of the printed polymer devices, the number of fabricated unit cells within the devices for GT1, GT2, and GT3 was 25 × 25 × 36, 25 × 25 × 18, and 25 × 25 × 12 (L × W × H), respectively. Then, a 100 nm TiO_2_ shell was deposited onto the polymeric scaffold via atomic layer deposition (Figure 3C). This thickness was thin but strong enough to prevent collapse or cracking during high‐temperature treatments. Subsequently, the boundary surfaces of the printed devices were milled via focused ion beam to expose the interior polymer (Figure 3D). The sample was then subjected to a controlled thermal treatment in air for calcination of the polymeric scaffold, retaining the TiO_2_ tubular structure, whose EDS analysis is shown in Figure S9. This procedure ensures the complete removal of the polymer template (Figure 3E) while serving as a high‐temperature annealing stage for TiO_2_, which further enhances its dielectric constant (Figure S10). Finally, to increase the volume fraction of high dielectric materials, an additional 100 nm TiO_2_ was infiltrated into both the interior and outer surfaces. All the devices were fabricated on the same Si substrate (Figure 3F) to minimize the influence of the substrate in subsequent optical measurements.

The fabricated photonic crystal devices, GT1 to GT3, were characterized under an infrared microscope to collect their infrared spectra (Figure S11), and the obtained spectra are shown in Figure 3G. All three devices exhibit high reflectivity at 4.25 µm, with the reflectivity increasing progressively from 60.96% to 66.13% and 69.46%. At different sample rotation angles, the measured reflectance consistently exhibits a progressive increase from GT1 to GT3 (Figure S12). The monotonic increase in experimental reflectance had a great agreement with the band structure analysis of the three structures. When electromagnetic waves with frequencies within the PBG propagate through the photonic crystal, they undergo exponential spatial decay. The attenuation rate reaches a maximum as the frequency approaches the PBG center [40]. Since the fabricated photonic crystals possess a finite size rather than infinite periodicity, a portion of the incident light within the PBG may transmit through the structure. A wider bandgap results in more rapid evanescent decay, yielding superior optical confinement and higher reflectance. To further verify the robustness of this approach and exclude potential fabrication‐induced effects on the measured results, further independent batches of samples were fabricated, which exhibited the same reflectance trend as above (Figure S13).

Leveraging this experimental phenomenon, these architectures can be applied in advanced fields such as passive radiative cooling. The monotonic tunability of reflectance within a fixed frequency regime can correspond to high, medium, and low cooling power densities. The fact that both the fabrication scale and the dielectric materials remain unchanged means that the established process flow is compatible across different performance requirements, providing advantages for the streamlined and scalable industrial manufacturing of next‐generation photonic devices.

### 2.4 Mechanism Analysis

The characteristics of PBG are closely related to the distribution of electric‐field energy at different bands. In photonic crystals, the lower frequency modes tend to localize their electric field energy within the high‐dielectric regions. Moreover, owing to the Hermitian nature of the Maxwell eigenvalue problem, the eigenmodes in the upper band remain orthogonal to those in the lower frequency bands [40]. Therefore, the band below the PBG, usually called the Dielectric Band (DB), concentrates most of its energy within high‐dielectric regions, while the band above the PBG, known as the Air Band (AB), exhibits more energy localization in the low‐dielectric regions. The spatial distribution of the energy‐concentrated regions and the extent of localization have significant influence on the width and spectral position of the PBG [4, 5]. Here, we calculated the time‐averaged electric‐field energy density of DB of GT1 to GT3 at Γ *k*‐point (0,  0,  0) to study the mechanism of the PBG phenomenon, which of the other bands shown in Figures S14–S16. To facilitate comparison of the energy distributions among GT1 to GT3, the normalized electric‐field energy density used is defined as the ratio between local voxel energy value to the maximum voxel energy value within each system, details are provided in the Field analysis section of the Methods. The Γ *k*‐point was selected because the Bloch phase factor is unity at the Γ *k*‐point and the electric field distribution directly reflect the global energy profile of the mode.

GT1, GT2, and GT3 exhibit different electric field distribution patterns (Figure 4). For the tubular network‐like photonic crystals, multiple interconnected nodes and corresponding struts connecting them provide effective pathways to guide and redistribute the electric field throughout the dielectric network. In the design and fabrication of GT1 to GT3 structures, the additional mirror symmetry changes the gyroidal networks with only f3 nodes into tubular networks with f3 and f4 nodes, as mentioned above. Moreover, due to the mirror symmetry, the orientation of the electric field close to the mirror plane must be parallel to the plane. For GT1, all the nodes are located at the repeated mirror planes, and the electric field mainly converges in the struts near f4 nodes. Meanwhile, the repeated mirror planes are closely spaced and principally identical, making the converging electric field cannot be efficiently spread through the branching pathways (GT1 in Figure 4A). This leads to a discrete field pattern, which can also be directly reflected in the field energy distribution of the structure (GT1 in Figure 4B). As for GT2, with more gyroidal features preserved, the electric field can flow from the mirror planes into the gyroidal‐like portion, and the gyroidal f3 nodes can guide the electric field to spread into the network (GT2 in Figure 4A,B). But notable, in this situation, there is still restriction region of electric‐field distribution within the structure (GT2 in Figure 4A). For GT3, it has more gyroidal f3 nodes to guide the electric field into a more uniform and continuous distribution throughout the structure (GT3 in Figure 4A,B). GT1, GT2, and GT3 also have different normalized electric‐field energy density value histogram profiles (Figure S18). GT1 exhibits a highly nonuniform distribution. GT3 displays a more homogeneous distribution compared with GT1. GT2 shows an intermediate case between GT1 and GT3.

**FIGURE 4:**
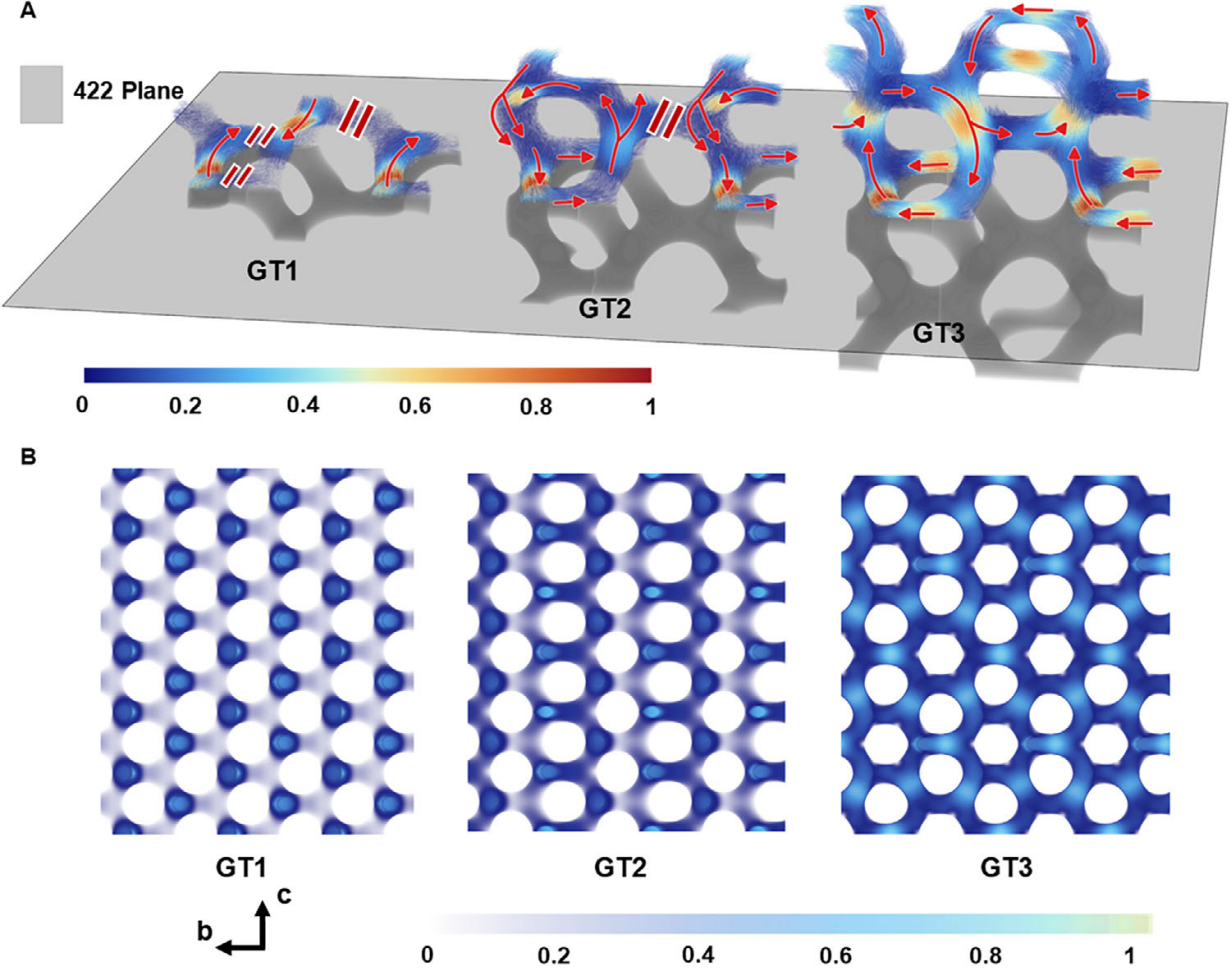
Electric‐field and energy analyses of the dielectric band (DB) at the Γ point of GT1 to GT3 photonic crystals. (A) Normalized electric‐field vectors and main electric‐field streams in GT1 to GT3 tubular networks. Electric‐field vector glyphs are plotted as lines only inside the dielectric region. The glyph color represents the normalized electric‐field energy density. The “||” exhibits a restriction of the electric‐field distribution in this region. (B) Normalized electric‐field energy density distribution in GT1, GT2 and GT3 viewed along the $\vec{c}$ direction. The color represents the value of normalized electric‐field energy density. The other views are shown in Figure S17.

Photonic crystals with more uniformly distributed electric field in the high‐dielectric region generally have wider photonic bandgaps [5]. As the electric‐field distribution patterns and electric‐field energy density value histogram profiles discussed above show, the distribution of electrical field from GT1 to GT3 changes from a nonuniform and discrete mode into a uniform and continuous mode gradually, resulting in a monotonic increase in the complete PBG width from GT1 to GT3. Meanwhile, the periodic twin mirror features as well as the similar morphology in GT tubular networks lead to analogous electric‐field propagation and similar electric‐field distribution patterns. And the identical volume fraction of networks and material composition minimize variations in the overall dielectric distribution. These factors work together to remain the central frequency of the PBG at a stable region.

## 3 Conclusion

The design of structures with different geometry, topology, and symmetry is the key basis for further development of photonic crystals with breakthrough properties. Structural defects, as deviations from the ideal, perfectly periodic structures, could provide a rich origin of new morphologies and symmetries. Here, based on the clear understanding of gyroid twin boundary morphology, we designed and constructed a series of gyroid twin tubular networks. Both calculations and experimental measurements demonstrated that they are good photonic crystal candidates. In addition, for this set of gyroid twin structures, by fine‐tuning the gap between two periodic twin mirror planes, monotonic tunability of the width of the PBG with a stable central frequency can be achieved. This study opens the door to introducing symmetries into classical structures to achieve extraordinary photonic performance based on structural defect engineering.

## 4 Methods

### 4.1 Generate Gyroid‐Twin Structure

The original gyroid structure is given by the level‐set equation as below

sin (2π*x*)*cos (2π*y*) + sin (2π*y*)*cos (2π*z*) + sin (2π*z*)* cos (2π*x*) =  *t*.

To generate the epsilon files that the photonic band calculation requires and the stl files for the print, we used Mathematica, where the equation was modified for twinning by the (422) plane of the single gyroid structure. We rotated the structure $\mathrm{acos}(\frac{1}{\sqrt{6}})$ degree around the axis (1, ‐2, 0) first, then $\mathrm{acos}(\frac{2\sqrt{2}}{5}-\frac{\sqrt{3}}{5})$ degree around the axis (0, 0, 1) to gain the unit cell of GT1 to GT3. Through the adjustment of *t* value, the structure epsilon files of different volume fractions can be obtained.

### 4.2 Photonic Band Structure Calculation

The calculation is carried on the open‐source software MPB, with the plane‐wave expansion method (PWE). To get smooth and accurate bands, we interpolate 10 points between the *k*‐points on the boundary of the irreducible orthorhombic Brillouin zone, while the redundant paths are exhibited in Figure S3. The number of bands calculated was 20. The resolution was set to 80, which is high enough to get precise calculation results. The mesh size was 3 by default. The calculation results are expressed as normalized frequencies.

### 4.3 Sample Preparation

The polymer scaffold of the samples was prepared through TPL on the Nanoscribe Quantum X platform. The resin used was IP‐DIP2, which is suitable for the micro print set. Moreover, a 2‐photon‐polymerized IP‐DIP2 structure can be completely removed through calcination at 600°C in air. The resin was dripped on the polished silicon substrate, which had been silanized, and the 63x print head was immersed in it for printing. The stl files of GT1 to GT3 were imported in the DeScribe software and sliced at 0.1 µm for the accuracy of the printed structure. The laser power and scan speed were set as 25 mW and 65 000 µm/s. After printing, the sample was submerged into propylene glycol methyl ether acetate (PGMEA) solvent for 10 min, to clean the unpolymerized resin and to develop the 3D‐printed structure. Meanwhile, the sample was irradiated with UV light to accelerate the development of the printed structure and reduce shrinkage during the process. Then the sample was submerged in the isopropanol solvent for 8 min to finish the development and clean the PGMEA with the remaining resin.

The ALD system (Beneq TFS 200) was used to conformally grow TiO_2_ onto the polymer scaffold at 200°C. The H_2_O and TiCl_4_ were used as precursors, both with a pulsing time of 0.15s. After 2000 cycles, a 100nm TiO_2_ coat can be obtained. Then the boundaries of the structure were milled using FIB (Thermo Scientific Scios 2 DualBeam). The voltage and current of the ion beam were set as 30.00kV and 3.00nA. After exposure of the polymer scaffold, the sample was placed in the tubular furnace (OTF‐1200X, Hefei Kejing), where the temperature was slowly ramped to 600°C in an hour and held for 2 h to remove the polymer completely. Moreover, the tubular structure was treated with the same ALD process to grow another 100nm TiO_2_ both onto the surface and into the interior. SEM (Thermo Scientific Scios 2 DualBeam) was used to image the samples in the process, with an electron beam whose voltage and current were set to 10.00 kV and 0.80nA.

### 4.4 IR Spectra Acquisition

The reflection spectra were measured on the Fourier Transform infrared spectroscopy (PerkinElmer Spotlight 200i). The cassegrain would focus the infrared light from the source, then irradiated toward the sample with an angle range of 15°–36°, and the reflected light would be collected by an MCT detector. Reflection spectra were acquired from a 30 µm × 30 µm aperture region at the center of the sample. Therefore, the collected spectra were a weighted average of the reflected signal over this angular range. The monotonic bandgap width modulation of GT1 to GT3 is primarily manifested in the complete photonic bandgap. The reflectance spectra acquired in this way can effectively reveal the omnidirectional PBG.

### 4.5 Field Analysis

The time‐averaged electric‐field energy density ε|**E**|^2^ of different bands at the *k*‐points was gained through the MPB output_dpwr function. The output electric field (**E**) is normalized by the function as below,

$$\int {\left(\varepsilon {\left|E\right|}^{2}\right)}_{i}dV_{i}=1$$

where the subscript *i* denotes the *i*‐th grid voxel, (ε|**E**|^2^)_***i***_ represents the local electric‐field energy density at the *i*‐th grid voxel, *dV_i_* is the volume of the *i*‐th grid voxel, and the integrals are over the computational cell. Therefore, to facilitate visualization and comparison of the spatial energy distributions among different systems, a normalized electric‐field energy density is further defined as

$${\left(\varepsilon {\left|E\right|}^{2}\right)}_{normal,i}=\frac{{\left(\varepsilon {\left|E\right|}^{2}\right)}_{i}}{\max_{i}\left(\varepsilon {\left|E\right|}^{2}\right)}$$

where the denominator represents the maximum electric‐field energy density among all grid voxels within the same system. The normalization highlights the relative spatial distribution, localization, and uniformity of the electric‐field energy in different structures. The normalized electric‐field energy density was then visualized on Paraview.

## Author Contributions

**Shuo Huang**: writing – original draft, investigation, methodology, validation, formal analysis, visualization, data curation. **Yang Zhou**: methodology, investigation, validation, visualization. **Wenxin Ning**: investigation, methodology, validation, visualization. **Hengliang Wang**: methodology, investigation. **Lu Han**: writing – review and editing, funding acquisition, formal analysis. **Xueyan Feng**: conceptualization, methodology, data curation, formal analysis, supervision, funding acquisition, project administration, resources, writing – review and editing.

## Funding

This work was supported by the National Natural Science Foundation of China, the Excellent Young Scientists Fund Program (X. F.), 22425303 (L. H.).

## Supporting information




**Supporting File**: advs78055‐sup‐0001‐SuppMat.docx.

## Data Availability

All the study data are included in the article and supporting information.
