# Supplementary material for: Defect‐Symmetry Engineered Gyroidal Photonic Crystals Achieve Monotonic Modulation of Bandgap Width With a Stable Mid‐Gap
Source: Adv Sci (Weinh). 2026 Sep 29:e78055. Online ahead of print. doi: 10.1002/advs.78055 (PMC13624417; doi:10.1002/advs.78055)
Supplement: Supplementary file 1 [file ADVS-9999-e78055-s001.docx]

Supporting Information

Defect-symmetry engineered gyroidal photonic crystals achieve monotonic modulation of bandgap width with a stable mid-gap

Shuo Huang^1^, Yang Zhou^1^, Wenxin Ning^1^, Hengliang Wang^2^, Lu Han^3^, Xueyan Feng^1^*

^1^State Key Laboratory of Molecular Engineering of Polymers, Department of Macromolecular Science, Fudan University; Shanghai 200438, China.

^2^Department of Physics, Fudan University; Shanghai 200438, China.

^3^School of Chemical Science and Engineering, Tongji University, Shanghai 200092, China

*Corresponding author. Email: fengxueyan@fudan.edu.cn

**Figure S1. 3D experimental reconstruction of the twin grain boundary defect within a block copolymer double gyroid networks.** One of the interpenetrating gyroid networks are highlighted.

**Figure S2. The relationship between the volume fraction of the model and the** $\boldsymbol{t}$ **value.** The gyroid segments between adjacent (422) planes are presented. As the $t$ value increases, the volume fraction continuously decreases.

**Figure S3. Smallest loop analysis of SG and GT1 to GT3.** Each loop was classified by the total number of nodes and the functionality of the nodes along the path; for example, the ideal gyroid smallest loop is denoted as 10 - 3^10^, corresponding to a closed path of ten three-connected nodes.

**Figure S4. The periodicity on the (422) plane of SG.** The structure is periodically arranged along the $[1\bar{1}\bar{1}]$ and $[01\bar{1}]$ directions, with corresponding periodicities of $\frac{\sqrt{3}}{2}l$ and $\sqrt{2} l$, respectively.

**Figure S5. The photonic band structures of redundant paths along the boundary of irreducible orthorhombic Brillouin zone of GT1 to GT3.**

**F****igure S6. The unit cells and photonic band structures of GT4 to GT6.** The SG parts between two parallel twin planes with different gap distances of $4\times\frac{\sqrt{6}}{6}l$, $5\times\frac{\sqrt{6}}{6}l$, $6\times\frac{\sqrt{6}}{6}l$ are cropped out and mirrored periodically to form the GT4, GT5, GT6 tubular network structures, respectively. And their unit cells are shown as above. All GT4 to GT6 structures exhibit complete photonic bandgaps.

**Figure S7. The unit cells, Brillouin zone and photonic band structures of SG.** The left panel shows the band structure of standard Brillouin zone of the SG structure. To facilitate comparison with the GT photonic crystals, the right panel presents the band structure calculated using a unit cell oriented along the [211] direction. The $t$ value was set to 0.9, consistent with that used for the GT structures.

**Figure S8. Comparation of the fabricated GT1 to GT3 photonic devices and designed structures.** The rendered pictures of models exhibit the characteristic faces of GT1 to GT3. The SEM images of milled fabricated GT1 to GT3 photonic devices show a great agreement with the models.

**Figure S9. EDS analysis of GT1 photonic device.** After complete remove of the polymer template, TiO_2_ hollow structures remained. The EDS mapping confirms that the elemental distribution in the sample is consistent with the expected composition.

**Figure S10. The optical constants of TiO_2_ from ALD deposition.**

**Figure S11. Schematic illustration of the infrared spectral acquisition setup.** The light from source was focused by cassegrain and irradiated towards the sample with an angle range of 15°-36°. And the reflected light was collected by the detector.

**Figure S12. Reflectance spectra measured at different rotation angles.** After rotating the samples by 0 ° (A), 45 ° (B) and 90 ° (C), the reflectance exhibits a progressive increase from GT1 to GT3.

**Figure S13. Two independently fabricated samples and their corresponding measured reflectance spectra.** Across both fabrication batches, the reflectance consistently increases from GT1 to GT3. Scale bar: 50 μm.

**Figure S14. The normalized electric fields distribution of GT1 at the Γ point at different bands. The color represents the normalized electric-field energy density.**

**Figure S15. The normalized electric fields distribution of GT2 at the Γ point at different bands.** **The color represents the normalized electric-field energy density.**

**Figure S16. The normalized electric fields distribution of GT3 at the Γ point at different bands.** **The color represents the normalized electric-field energy density.**

**Figure S17. Other views of electric-field energy distributions of the dielectric band (DB) at the Γ point of GT1 to GT3 photonic crystals.** GT1 exhibits a discrete electric-field energy distribution pattern. For GT3, the electric-field energy shows a more uniform and continuous distribution throughout the gyroidal structs. GT2 shows an intermediate case between GT1 and GT3. The color represents the normalized electric-field energy density.

**Figure S18. Comparison of the distribution histograms of normalized electric-field energy density for GT1-GT3.** GT1 exhibits a highly nonuniform distribution. GT3 displays a more homogeneous distribution compared with GT1. GT2 shows an intermediate case between GT1 and GT3.
